# Crystallographic Structure and Antiglioma Potential of *Centrolobium microchaete* Seed Lectin

**DOI:** 10.1021/acsomega.4c09145

**Published:** 2025-01-27

**Authors:** Benildo Sousa Cavada, Vanir Reis Pinto-Junior, Francisco Edilcarlos Oliveira Lima, Valeria Maria Sousa Ferreira, Messias Vital Oliveira, Vinicius Jose Silva Osterne, Nicole Sartori, Ana Carolina dos Santos, Rodrigo Bainy Leal, Kyria Santiago Nascimento

**Affiliations:** † Department of Biochemistry and Molecular Biology, BioMolLab, Federal University of Ceara, Fortaleza 60020-181, CE, Brazil; ‡ Laboratory of Biochemistry and Glycobiology, Department of Biotechnology, Ghent University, 9000 Ghent, Belgium; § Department of Biochemistry and Postgraduate Program in Biochemistry, Center for Biological Sciences, University Campus, Federal University of Santa Catarina, Florianópolis 88040-900, SC, Brazil

## Abstract

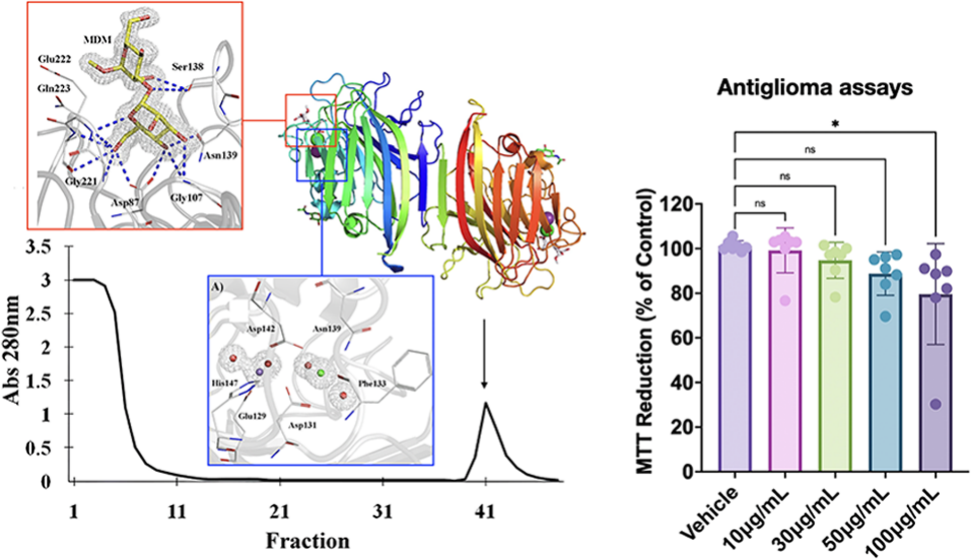

The genus *Centrolobium* comprises species of Neotropical trees with seeds that possess medicinal and bioactive applications. Lectins from this genus exhibit anti-inflammatory and immunomodulatory effects, influencing the activation of the immune system. This study focuses on characterizing the structure and carbohydrate-binding properties of the lectin from *Centrolobium microchaete* (CML) and evaluating its potential against gliomas. The structure of the lectin in complex with methyl-mannose-α1,3-mannose (MDM) was resolved using X-ray crystallography at 1.3 Å resolution, with its interactions further analyzed through molecular dynamics simulations. Structurally, CML adopts a β-sandwich motif and assembles into canonical dimers. *In vitro* assays revealed that CML reduced the viability of C6 glioma cells, although only at high concentrations, without impacting cell migration or morphology. CML activated autophagic processes, albeit with lower efficacy compared with other mannose-specific lectins. The limited antiglioma activity of CML may be linked to its inability to form tetramers and unusual specificity toward asymmetric glycans, both crucial features for interactions with cellular glycans and the activation of signaling pathways. This study represents the first investigation of the antiglioma potential of a mannose-specific lectin from the Dalbergieae tribe, highlighting both its structural characteristics and functional limitations.

## 1 Introduction

The genus *Centrolobium* from the *Leguminosae* family, is a Neotropical group with seven species. These are primarily large trees that can reach up to 30 m in height, featuring a straight trunk and a wide, rounded crown of terminal branches. The fruits are large, winged samaras up to 30 cm long, and feature a conspicuous spiny seed chamber at the base. Several species of *Centrolobium* are valued in the Neotropics for their durable wood, which exhibits a rich yellowish-orange color with patches of dark red, purple, or black. Taxonomic studies indicate that the genus *Centrolobium* includes the species *C. microchaete*, *C. ochroxylum*, *C. paraense*, *C. robustum*, *C. sclerophyllum*, *C. tomentosum*, and *C. yavizanum*. These specimens grow primarily in areas of dry seasonal tropical forests, but can also be found in wetter forests. The genus is distributed across parts of Brazil, Bolivia, Ecuador, Peru, Colombia, Venezuela, Panama, and the Guianas.^1,2^

Ethnobotanical knowledge about the species *C. microchaete* reveals the use of its seeds for medicinal, food, and agricultural purposes. The seeds are used in traditional medicine to treat inflammation and pain. Traditional communities prepare infusions from the seeds to apply to wounds and skin infections, making use of their antimicrobial activity. Several indigenous communities in the Amazon use *C. microchaete* seeds in both medicinal and agricultural practices. In regions of the Brazilian Cerrado, the seeds are employed in home remedies and for the restoration of degraded pastures. The biochemical composition of *C. microchaete* seeds has been extensively studied, revealing a variety of bioactive compounds. These compounds include fatty acids, proteins, carbohydrates, and alkaloids. The seeds are also rich in essential minerals, such as calcium, iron, and zinc, and contain significant amounts of phenolic compounds, powerful antioxidants with high potential for pharmaceutical and nutraceutical applications

Studies on *C. microchaete* seeds reveal the presence of a lectin called CML. This lectin binds mannosides and glucosides and can agglutinate rabbit erythrocytes. CML shares physicochemical properties with other lectins from the same genus, including those from *C. tomentosum* (CTL) and lectins from the Dalbergieae tribe.^3^ The structure of CML can be characterized as a glycosylated protein with two metal-dependent isolectins. Biological studies demonstrate that both CML and CTL induce dose-dependent paw edema and stimulate leukocyte migration to peritoneal cavities.^3^ The presence of α-methyl-D-mannoside mitigates the inflammatory effect of CML, indicating a dependency on sugar binding for the observed effects. *In silico* studies by Neco et al. employed homology modeling to characterize the three-dimensional structure of CML, highlighting its protein–carbohydrate interactions and their link to the inflammatory effect.^4^ CML was also able to accelerate wound healing in mice, promoting a faster and more complete restructuring of the skin. The treatment proved to be effective and promising for use in acute wounds.^5^

Gliomas are the most common primary brain tumors. Among these, glioblastoma multiforme (GBM; grade 4 astrocytoma) notable for its high malignancy, characterized by its proliferative and infiltrative properties, as well as its the ability to develop resistance to available treatments.^6^ Hence, GBM prognosis is unfavorable with an average patient survival of approximately 15 months after diagnosis. It is well-recognized that cancer cells display alteration of the glycosylation pattern on their cell surface.^7^ This phenomenon has even been exploited to induce selective cell death in some types of tumors.^8^ Glioblastomas also present modifications in the structure and composition of the glycans that decorate the cell surface.^9,10^ This property can be exploited to develop tools to study glioma biology or therapeutic strategies to counteract tumor progression.^11^ In a carbohydrate-recognition domain (CRD)-dependent manner, some legume lectins can modulate several cell signaling pathways, induce autophagy and apoptosis, and reduce cell viability and migration in gliomas.^11−14^ We recently demonstrated that the mannose-specific lectin of *Canavalia brasiliensis* (ConBr) can interact with glycans of the regulatory subunit of Xc- system, a membrane amino acid antiporter, responsible for cystine/glutamate exchange. This interaction inhibits glutathione synthesis, promoting oxidative stress and inducing cell death in glioma cells of the C6 lineage.^15^ Therefore, it is very important to characterize the structure of novel lectins, evaluate their antiglioma potential and establish a comparison with the antitumor properties of other lectins.

The specificity of lectin–carbohydrate interactions is crucial to determining the biological functionality of lectins. Research on *C. microchaete* lectin has investigated its binding specificity to wards various carbohydrates structures. Delving deeper, structural studies, including X-ray crystallography, allow the elucidation of the three-dimensional conformation of CML, revealing further details of its interactions with carbohydrates. These insights being vital for understanding the mechanisms of action of lectins. Considering the aforementioned, this study aimed to obtain the structural properties of CML using crystallographic techniques and *in silico* glycan interaction analyses. The findings are expected to contribute significantly to lectinology, providing valuable insights into the structural features and molecular interactions of CML. Furthermore, the investigation of the biological activity against a glioma cell model could provide valuable insights into the antiproliferative properties of legume lectins.

## 2 Materials and Methods

### 2.1 CML Purification and Sample Preparation for Crystallization Assays

CML has been purified following the protocol from Vasconcelos et al. with modifications.^3^ Briefly, soluble proteins from the defatted seed flour was extracted at a ratio of 1:10 in 100 mM Tris-HCl buffer, pH 7.6, containing 150 mM NaCl, under constant stirring for 4 h at room temperature. Subsequently, the crude extract was centrifuged at 9000*g* for 20 min at 4 °C. The supernatant was collected and filtered through Whatman filter paper (grade 1/11 μm) and the clarified extract was then applied to a column (1.0 × 3.5 cm^2^) containing a pre-equilibrated agarose–mannose matrix. The fraction eluted with the equilibration buffer was discarded, and the retained fraction containing (CML) was eluted with 100 mM D-mannose in the equilibration buffer. Fractions of 1.5 mL were collected manually and monitored by spectrophotometry at 280 nm wavelength. An AMICON Ultra 15 mL system (NMWL 10 kDa) was used to concentrate and desalt the active fractions. The sample was initially concentrated and then treated with 100 mM sodium acetate, pH 4.0, through four centrifugations cycles at 5000*g* for 20 min at 4 °C to remove bound D-mannose. The sample then underwent four additional centrifugations to exchange the buffer to 25 mM Tris-HCl buffer, pH 7.6. Finally, the CML sample was concentrated until approximately 10 mg/mL. Concentration was assessed using the Bradford method.^16^

### 2.2 CML Crystallization

The crystallization test was carried out using the vapor diffusion method according to Jancarik and Kim.^17^ To this end, the sample was conditioned in 25 mM Tris-HCl at pH 7.6 at a concentration equal to, or above, 10 mg/mL and incubated with 5 mM MDM for 1 h at 37 °C. After centrifugation at 10,000*g* for 10 min, the supernatant was collected and used to assemble condition screening plates using JBScreen JCSG++ (Jena Bioscience) and Crystal Screen I, II and Index (Hampton Research) crystallization kits. Assembly of the initial screens was carried out with the aid of the Mosquito Crystal crystallization robot (TPP Labtech, Hertfordshire, UK), using the hanging drop method with a 96-well plate (SWISSCI) maintained at a temperature of 18 °C. After obtaining the initial condition, optimizations were perfomed by adjusting the concentration and pH of the crystallization agents. A 48-well MRC crystallization plate (SWISSCI AG) was prepared with 4 μL of sitting drops in a 1:1 ratio with saturated lectin solution and 200 μL of crystallization solution for each well in a closed system. The plate was incubated at a temperature of 37 °C until crystals suitable for X-ray diffraction experiments were obtained.

### 2.3 Collection and Processing of CML Crystallographic Data

Suitable crystals were incubated with the crystallization condition supplemented with 20% glycerol as a cryoprotectant. X-ray diffraction experiments were carried out at the MANACA Station of the National Synchrotron Light Laboratory (SIRIUS, LNLS-Campinas, Brazil), using a PILATUS 2M detector (Dectris, Switzerland). A total of 3600 images were collected, each with an oscillation angle of 0.1°, from a single crystal cooled to 100 K. Following, the data were processed using the XDS package (v. Jun 30, 2024), and the number of molecules per asymmetric unit was determined using the Matthews_Coef software.^18,19^ Data reduction was performed with the Scala v3.3.21 program from CCP4 v8.0.^20,21^ The crystal structure of CML was determined by molecular replacement using Molrep v. 11.0/22.07.2010/^22^ with coordinates of the lectin from *Centrolobium tomentosum* seeds (PDB id: 5EYY) adjusted to the amino acid sequence of CML (UniProt ID: C0HK20).^23^ The refinement, addition of water molecules and ligands, and generation of maps were carried out with PHENIX v. 1.21.^24^ When necessary, manual modifications were made using the WinCoot program v. 0.9.8.93.^25^ Model quality validation was carried out using the PDB Validation tool. Polar and van der Waals interactions were evaluated with the CONTACT program. The 3D and 2D figures were generated using the PyMOL Molecular Graphics System, Version 3.0 Schrödinger, LLC and LigPlot+ v. 2.2.^26^ The CheckMyMetal (CMM) server v 2.1 was used for validation and analysis of the metal coordination geometry (https://csgid.org/csgid/metal_sites/).^27,28^ The coordinates and structure factors have been deposited in the RCSB Protein Data Bank with Accession Code 9C4I.

### 2.4 Glioma Cell Culture and Treatments

C6 cells from Wistar rat glioblastoma (*Rattus norvegicus*) were acquired from a cell bank in Rio de Janeiro (Brazil). The cell culture procedure was performed as previously described.^14^ Briefly, cells were grown in 25 cm^2^ culture bottles with Dulbecco’s Modified Eagle’s Medium (DMEM) supplemented with 10% (v/v) fetal bovine serum (FBS) (Gibco), 100 units/mL penicillin, and 100 mg/mL streptomycin (Gibco) at 37 °C in a humidified atmosphere of 95% air and 5% CO_2_. The medium was changed every 2 days until the cells reached 80% confluence. Thereafter, cells were washed with phosphate-buffered saline (PBS) (140 mM NaCl, 3 mM KCl, 10 mM Na_2_HPO_4_ and 2 mM KH_2_PO_4_, pH 7.4) and dissociated with trypsin. The cell pellet was resuspended with 3 mL of medium, and the cell concentration was determined by counting in a Neubauer chamber. Subsequently, C6 cells were seeded in 6-, 24- or 96-well plates with DMEM medium supplemented with 10% (v/v) fetal bovine serum (FBS) (Gibco), 100 units/mL penicillin, and 100 mg/mL streptomycin (Gibco) for 24 h at 37 °C in a humidified atmosphere of 95% air and 5% CO_2_. Prior to treatment, the plates were observed under an inverted microscope to assess cell adherence and confluence. Afterward, the culture media were replaced with fresh media containing vehicle or CML at final concentrations of 10, 30, 50, or 100 μg/mL and incubated for 24 h. The lectin stock solution (2 mg/mL) was prepared by dilution of CML with HEPES-saline buffer without glucose (vehicle) composed of 500 mM 4 mM NaCl, KCl, 1.2 mM MgSO_4_, 25 mM HEPES, and 1 mM CaCl_2_, pH 7.4. For all assays, the control cell cultures were incubated with a vehicle (HEPES-saline buffer without glucose).

### 2.5 Determination of Cell Viability

Cell viability was assessed using the 3-[4,5-dimethylthiazol-2-yl]-2,5-diphenyltetrazolium (MTT) reduction method as previously described.^14^ In this assay, MTT is converted to a purple formazan, insoluble after cleavage of the tetrazolium ring by cellular dehydrogenases. The purple formazan is proportional to the cell viability. Briefly, the cells were seeded in a 96-well plate at a density of 10,000 cells/well and maintained for adhesion for 24 h. Thereafter, the culture medium (DMEM) was replaced with the same medium without FBS containing 0.2% albumin, and treatment over the course of 24 h was performed with the vehicle (control) or CML at 10, 30, 50, and 100 μg/mL. Upon completing the treatment period, the medium was removed, and cells were incubated for 1 h and 30 min at 37 °C with MTT dissolved in HBSS (Hank’s Balanced Salt Solution—saline buffer containing 136 mM NaCl, 5.4 mM KCl, 1.4 mM MgCl_2_·6H_2_O, 1 mM NaH_2_PO_4_, 1.2 mM CaCl_2_.2H_2_O, 10 mM HEPES, and 9 mM glucose, pH 7.4) for a final concentration of 0.5 mg/mL. Reduced MTT formazan crystals were dissolved with 100 μL of dimethyl sulfoxide (DMSO), and the absorbance was evaluated at 540 nm using the Tecan Microplate Reader Infinite M200 located in the Laboratório Multiusuário de Estudos em Biologia at the Universidade Federal de Santa Catarina (LAMEB/UFSC). The results were expressed as a percentage of the control/vehicle (considered as 100% viable). The values obtained through the absorbance reading were transformed into percentages of cell viability relative to the mean of cell controls, considered as equivalent to 100% of the viable cells. For this assay, seven distinct cell growths (*n* = 7) were used and measured in triplicate.

### 2.6 Cell Migration (Scratch Assay)

To evaluate the migration capacity of C6 cells in response to treatments, a migration assay was carried out, following the protocol originally described by Liang, Park, and Guan with adaptations.^14,29^ Briefly, C6 glioma cells were plated in a 6-well plate at a concentration of 500,000 cells per well. Cells were grown for 24 h until confluence, and then a wound was introduced in each well by scraping cell layers with a P200 pipet tip. Thereafter, the medium was removed, and serum-free DMEM containing 0.2% albumin with the vehicle (control) or CML (10–100 μg/mL) was added. For analysis, images were captured at the 0, 24, and 48 h treatment time points by an inverted NIKON eclipse T2000-U microscope. The wound closure was calculated by using ImageJ software. Cell migration was evaluated by measurement (as pixels) of the open space produced by the scratch, and it was expressed as the percentage of closure related to control cells considered as 100% closure (at time point 48 h). For this assay, three distinct cell growths (*n* = 3) were used.

### 2.7 Acridine Orange Staining of Acidic Vesicular Organelles

Acridine Orange (AO) assay was performed as described previously to evaluate acidic vesicular organelle (AVO) formation, which may arise because of increased autophagic process.^13,14,30^ Briefly, C6 cells were plated in a 12-well plate at a density of 50,000 cells per well. After 24 h, the culture medium was changed, and cells were treated for 24 h with a vehicle or CML (10, 30, 50, and 100 μg/mL). In order to compare the effect of CML with other mannose-specific lectins previously described to induce autophagy,^13^ C6 cells were also treated with ConBr at a concentration of 50 μg/mL. Evaluation of autophagy was performed by fluorescence microscopy using the Nikon Eclipse T2000-U inverted microscope using filter sets, 470 nm excitation and 525 nm emission for chromatin analysis (CR; green fluorescence), and 350 nm excitation and 615 nm emission for acidic vesicular organelles (AVO; orange/red fluorescence) detection. For this assay, three distinct cell growths (*n* = 3) were used, and a representative experiment was presented.

### 2.8 Western Blotting

The levels of LC3I/LC3II were evaluated by Western blot to characterize autophagy, as described previously by Leal et al. with modifications.^14^ For this purpose, 500,000 cells were plated per well in a 6-well plate for 24 h. Thereafter, glioma C6 cells were treated for 24 h with CML (30–100 μg/mL) or ConBr (30 μg/mL). After treatment, the cells were carefully removed with the aid of a cell scraper and homogenized with 100 uL of 4% SDS Stop Solution (50 mM Tris, 2 mM EDTA, 4% SDS, pH 6.8). After aliquots of the homogenate were collected for protein determination, the dilution buffer (40% glycerol, 100 mM Tris and bromophenol blue) was added in each sample in a ratio of 1:4. Next, 2-mercaptoethanol was added for a final concentration of 8%. Proteins were separated by sodium dodecyl sulfate-polyacrylamide gel electrophoresis (SDS-PAGE) using 16% separation gel and 4% stacking gel. Electrophoresis was performed with a fixed current of 15 mA/plate and a maximum voltage of 180 V for approximately 2.5 h using the upper (190 mM glycine, 25 mM Tris and 0.1% SDS) and lower (50 mM Tris; pH 8.3) buffers. After electrophoresis, the gels were subjected to electrotransfer using a semidry system (1.2 mA/cm2; 1.5 h), as described by Bjerrum and Heegaard.^31^ Thus, the membranes were transferred to the nitrocellulose membrane and then stained with Ponceau (0.5 wt % Ponceau in 1% acetic acid). For immunodetection, the membranes were washed with Milli Q water and then incubated for 10 min with “Antigen Pretreatment Solution” (Thermo Scientific, Cat # 46640). Afterward, the membranes were washed again with Milli Q water and incubated with “Blocking Buffer” (Thermo Scientific; Cat. No. 37536) for 1 h. Then, membranes were washed with TBST (0.1% Tween-20, 10 mM Tris, 150 mM NaCl, pH 7.5) and incubated for 1 h with anti-LC3 antibody (Cell Signaling, Catalog No. 12741; 1:1,000). β-actin was detected for load control using anti-β-actin antibody (Santa Cruz Biotechnology, Catalog No. sc-47778; 1:2,000). The primary antibodies were diluted using “Primary Antibody Diluent” (Thermo Scientific, Cat. No. 46640), while the secondary antibodies were diluted in TBST containing 2% bovine albumin and incubated for 30 min. Proteins were then detected by chemiluminescence using the SuperSignal West Pico PLUS Chemiluminescent Substrate (Thermo Scientific, Cat. No. 34577) according to the manufacturer’s instructions. Protein analysis was performed using a ChemiDoc Photodocumentation device (Bio-Rad), which was available at the Multiuser Laboratory of the Federal University of Santa Catarina (LAMEB).

## 3 Results and Discussion

### 3.1 CML Structure

**Figure 1:**
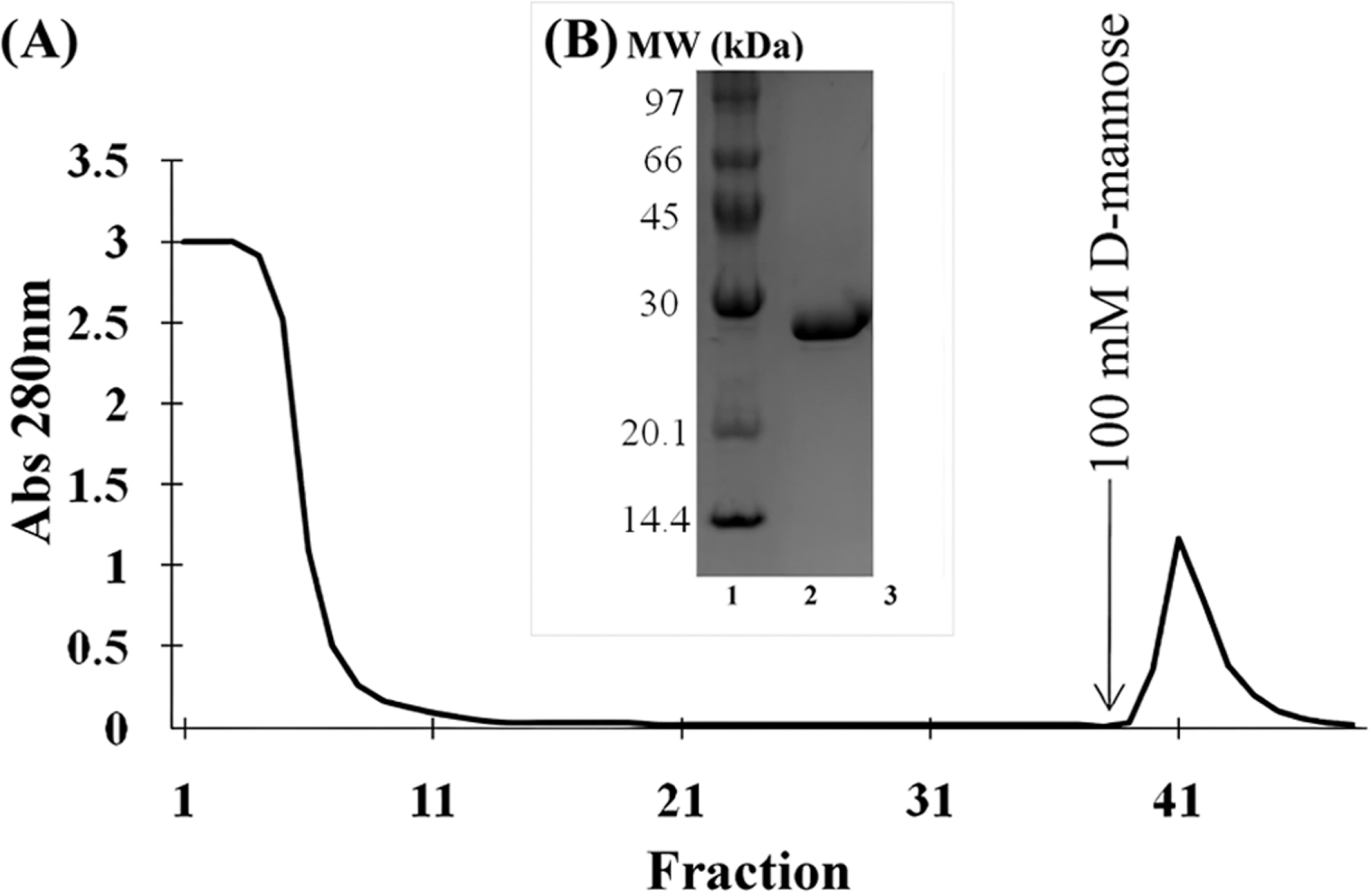
(A) Purification of *C. microchaete* lectin (CML) by affinity chromatography: 3 mL of crude extract was applied to an agarose/mannose affinity chromatography matrix (dimensions 5 × 2 cm^2^) for CML purification. The unretained fraction was eluted with 100 mM Tris-HCl buffer with 150 mM NaCl, while CML was eluted with 100 mM Tris-HCl, 150 mM NaCl, and 100 mM D-mannose. Fractions of 1.5 mL were collected with a flow rate of 1 mL/min. (B) SDS-PAGE: line 1—molecular markers: phosphorylase b (97 kDa), bovine serum albumin (66 kDa), ovalbumin (45 kDa), carbonic anhydrase (30 kDa), trypsin inhibitor (20.1 kDa) and α-lactalbumin (14.4 kDa); line 2—CML (48 μg).

CML was purified in a single chromatographic step, as described by Vasconcelos et al. (Figure 1A).^3^ The sample presented an electrophoretic profile corresponding to that of pure lectin, with a relative mass close to 30 kDa (Figure 1B).

CML crystals were obtained in a crystallographic conditions containing 200 mM ammonium sulfate, 100 mM HEPES, pH 7.5, and 25% PEG 3350. The diffraction data of the crystal indicated that it belongs to the monoclinic space group C121 (Table 1).

**Table 1:** Data Collection, Refinement, and Structure Quality Parameters

PDB ID 9C4I	
parameters	values
data collection	
space group	C 1 2 1
unit cell parameters	146.62; 41.98; 94.76 Å
	90.00; 102.72; 90.00°
number of reflections	276,434 (27,513)^d^
number of unique reflections	138,969 (19.912)^d^
molecules per asymmetric unit	2
resolution limits	46.22–1.3 (1.37–1.3)^d^
*R*merge^a^ (%)	5.1 (53.2)^d^
completeness (%)	99.77 (98.40)^d^
CC1/2	99.9 (94.9)^d^
multiplicity	6.6 (6.3)^d^
average I/σ(I)	17.3 (3.4)^d^
Wilson B-factor (Å^2^)	11.3
refinement	
resolution range	43.42–1.3
*R*factor^b^ (%)	17.33 (28.97)^d^
*R*free^c^ (%)	19.06 (31.68)^d^
number of reflections	138,823 (13,735)^d^
reflections used in *R*free	6934 (675)^d^
number of residues in asymmetric units	482
number of water molecules	758
variations of RMS optimal values	
bond length (Å)	0.007
bond angles (degree)	1.33
temperature factors	
average B-factor (Å^2^)	19.37
ligand	32.15
solvent	31.37
rotamers and Ramachandran plot	
rotamer outliers (%)	0
residues in most favored regions (%)	97.49
residues in additional allowed regions (%)	2.51
residues in generously allowed regions (%)	0

a .

b .

c *R*free was calculated using a random subset of 5% of reflections excluded from refinement.

d Values in parentheses represent the OuterShell.

Based on a mass of approximately 27,286 Da and 247 residues of the CML monomer (UniProt ID: C0HK20), the calculated Matthews coefficient was 2.68 Å^3^ Da^–1^, indicating the presence of one dimer per asymmetric unit and a solvent content of 54.05%. The CTL monomer (PDB ID: 5EYY) was chosen for molecular replacement, presenting a correlation score of 69% with a root mean square deviation (RMSD) of 0.227 Å for 193 Cα atoms when compared to the CML coordinates. Evaluation using the Ramachandran graph showed 97.49% of the residues in favorable regions, 2.51% in permitted regions, and no outliers, demonstrating that the model has acceptable stereochemistry.

**Figure 2:**
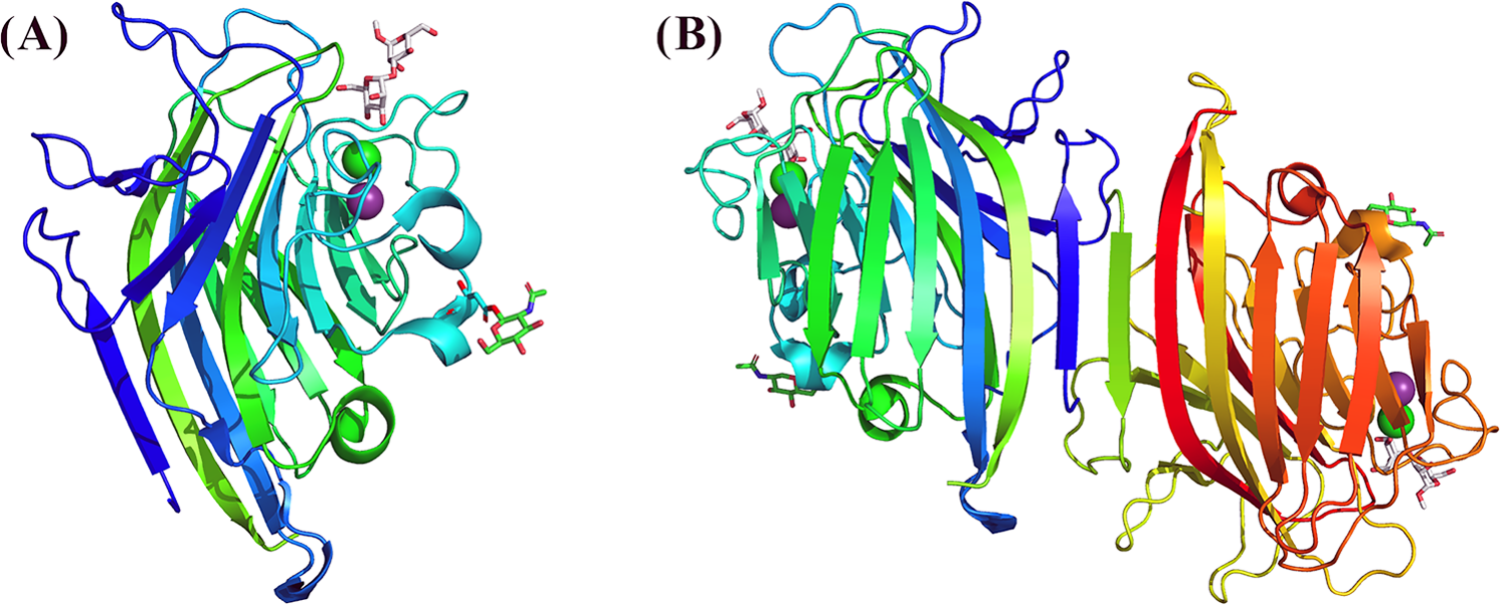
Overall structure of CML complexed with methyl-mannose-α1,3-mannose (MDM). (A) CML monomer; (B) canonical dimer of CML. Protein chains are represented as cartoons with different colors. MDM and *N*-glycosylation (GlcNAc) are represented as sticks with carbons in gray and green, respectively. Divalent cations are represented as spheres with calcium colored in green and manganese colored in purple.

Complexed with MDM, the CML structure was refined to a maximum resolution of 1.33 Å and a minimum of 46.22 Å, presenting a dimer with 482 amino acids, 2 MDM molecules, 2 calcium ions, 2 manganese ions, 2 *N*-acetyl-d-glucosamine molecules (NAG, from *N*-glycosylations), 5 sulfate molecules, 6 sodium ions, and 758 water molecules. CML monomers presented the *jellyroll* motif, or β-sandwich folding, which consists of two β-sheets: an extended rear of six strands and a curved front of seven strands connected by loops (Figure 2A). Each monomer contains a CRD, a metal-binding site (MBS) and an *N*-glycosylation (Figure S1). The dimer has a type II interface, also known as a canonical dimer, which consists of the formation of a larger β-sheet of 12 continuous strands, resulting from the interaction of two rear β-sheets of the monomers arranged side by side (Figure 2B). The dimeric structure was also confirmed by PISA (Protein Interfaces, Surfaces and Assemblies) analysis.^32^ The amino acids responsible for the formation and stabilization of the canonical dimer of CML are Ser1, Asp2, Ser3, Ser5, Phe6, Ser7, Ile9, Asp12, Glu15, Arg16, Asn17, Gln56, and Arg58 (Table 2 and Figure S2).

**Table 2:** Contacts between Amino Acids of Chains A and B at the Canonical Dimer Interface

chain A	chain B	distance (Å)
Ser1 OG	Glu15 OE2	2.65
Ser1 N	Glu15 OE1	2.66
Ser1 OG	Asn17 ND2	3.20
Asp2 OD2	Ile9 N	2.82
Asp2 N	Ser7 O	2.84
Ser3 OG	Ser7 OG	2.74
Ser5 O	Ser5 N	2.86
Ser5 N	Ser5 O	2.88
Ser5 OG	Ser5 OG	2.53
Ser5 CB	Ser5 OG	3.17
Ser5 OG	Ser5 CB	2.99
Phe6 CA	Ser3 O	3.16
Ser7 O	Asp2 N	2.88
Ser7 CB	Ser3 OG	3.15
Ser7 OG	Ser3 OG	2.67
Ile9 N	Asp2 OD2	2.80
Asp12 OD2	Arg58 NH1	2.64
Glu15 OE1	Ser1 N	2.74
Glu15 OE2	Ser1 OG	2.70
Arg16 NH1	Gln56 OE1	2.81
Asn17 ND2	Ser1 OG	3.05
Asn17 OD1	Gln56 N	2.92
Asn17 ND2	Gln56 O	2.87
Gln56 OE1	Arg16 NH1	2.87
Gln56 O	Asn17 ND2	2.85
Gln56 N	Asn17 OD1	2.91
Arg58 NH1	Asp12 OD2	2.80

### 3.2 Carbohydrate-Recognition Domain and Binding Analysis

**Figure 3:**
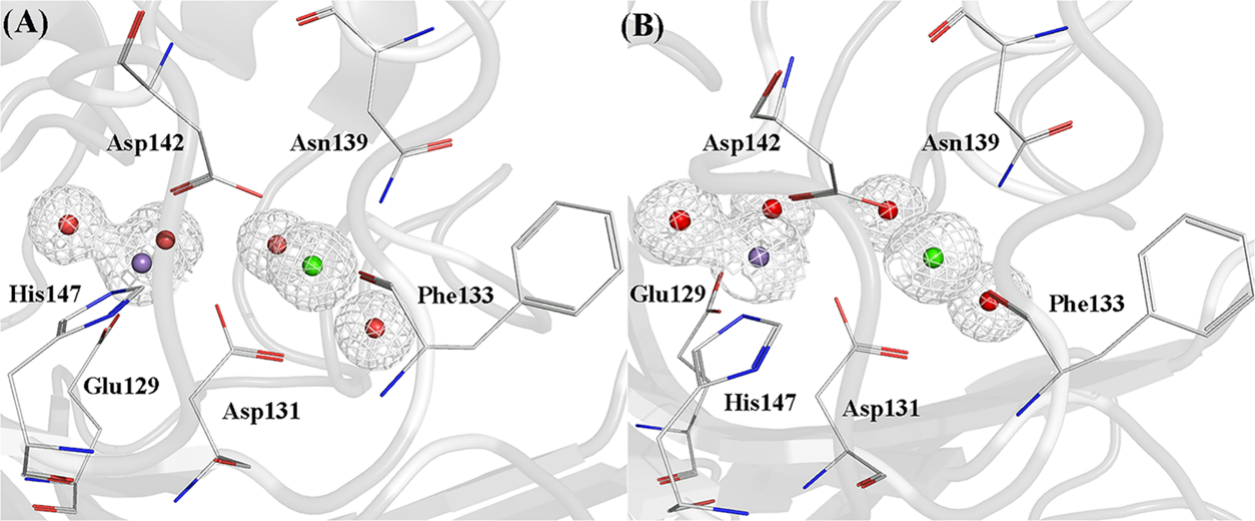
Representations of the metal-binding site in CML with the electron density map (2mFo-dFc) contoured at 1σ for Ca^2+^ and Mn^2+^. (A) Monomer 1 and (B) Monomer 2. Amino acids are represented in stick format with carbons in gray. Calcium ions are in green, manganese ions are in purple, and water molecules are in red, all represented as spheres.

Cocrystals of CML in the presence of MDM have been used in the current work to generate information about the structural basis of carbohydrate binding for this lectin. The electron density map revealed the presence of MDM in the two copies of CML found in the asymmetric unit, with the ligand’s relative location within each monomer matching what is expected for a legume lectin. In the MBS, the amino acids Glu129, Asp131, Asp142, and His147 coordinate the manganese ion, while Asp131, Phe133, Asn139, and Asp142 coordinate the calcium ion. Each ion is also associated with two water molecules (Figures 3 and S3). The presence of both cations was demonstrated by a CMM server analysis. The average B-factor of calcium ions was 10.5/10.7 Å^2^ and that of manganese ions was 16.6/11.2 Å^2^. Both presented 100% occupancy and an octahedral coordination geometry. As with other legume lectins, the calcium ion is responsible for stabilizing the *cis*-peptide between Ala86 and Asp87 through interaction with a water molecule, which is essential for the formation of the CRD.^33^

**Figure 4:**
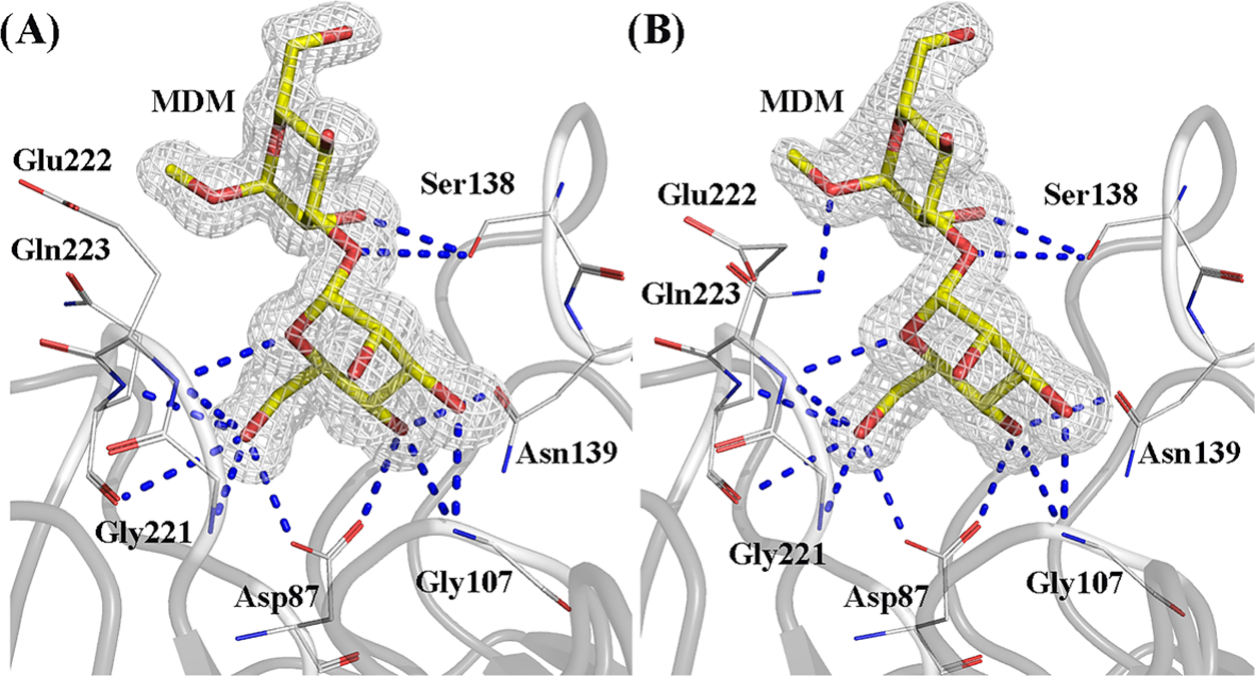
Carbohydrate-recognition domain of CML interacting with methyl-mannose-α1,3-mannose (MDM), while 2mFo-dFc omits a 1σ map representation around MDM. (A) Monomer 1 and (B) Monomer 2. Blue dashes represent polar contacts. Amino acids and MDM are represented as rod-shaped with carbons colored gray and yellow, respectively.

The MDM was stabilized in the CRD of CML by a hydrogen bond network involving atoms from the mannosyl group of the nonreducing end (MAN), methyl mannosyl group of the reducing end (MMA), and the amino acids Asp87, Gly107, Ser138, Asn139, Gly221, Glu222, and Gln223 of both monomers (A and B) (Figures 4 and S4). The interactions occurred with Asp87 such that the OD1 atom formed a hydrogen bond with MAN-O4 at a distance of 2.63 Å, and the OD2 atom bound to MAN-O6 at 2.72 Å. Gly107 A/B participated in the interactions through the N atom, binding to MAN-O3 at 2.87/2.85 Å and to MAN-O4 at 3.13/3.18 Å. Ser138 A/B contributed to its OG atom, forming a hydrogen bond with MMA-O2 at 2.85/2.88 Å and MMA-O3 at 3.29 Å. Asn139 A/B involved its OD1 atom in an interaction with MAN-O4 at a distance of 2.88/2.91 Å. Through its N atom, Gly221 A/B formed a bond with MAN-O6 at 3.25/3.22 Å. Glu222 A/B participated with its N atom, establishing bonds with MAN-O5 at 3.08/3.07 Å and MAN-O6 at 3.15/3.13 Å. Finally, Gln223 formed two interactions: one through the N atom with MAN-O6 at 3.07/3.05 Å and another through the O atom with MAN-O6 at 3.36/3.28 Å, and only Gln223-NE2 B formed a hydrogen bond with MMA-O1 at 2.58 Å.

The patterns observed in the MBS and CRD are similar to those observed for other mannose-specific lectins from the Dalbergieae tribe, such as the lectins from *Centrolobium tomentosum* (CTL), *Platypodium elegans* (PELa), and *Pterocarpus angolensis* (PAL), which have 95, 88, and 78% primary structure identity with CML and monomer RMSD of 0.227 Å (193 Cα atoms; PDB Id: 5EYY), 0.261 Å (204 Cα atoms; PDB Id: 5U38), and 0.342 Å (204 Cα atoms; PDB Id: 1Q8P), respectively (Figure S5).^23,33−35^

**Figure 5:**
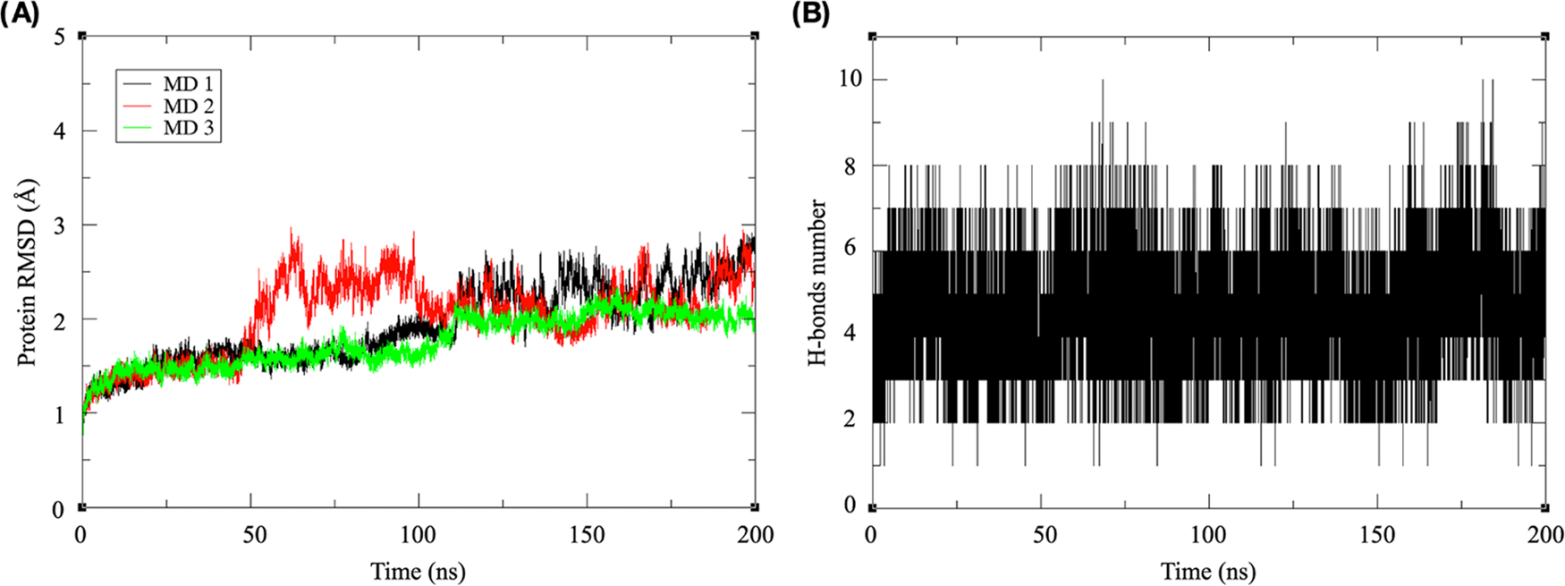
Molecular dynamics analysis. (A) Root mean square deviation (RMSD) plots. (B) Intermolecular hydrogen bonds between CML and MDM over the course of the simulation.

Binding was further studied through molecular dynamics (MD) simulations using the crystal structure as an input. Simulations ran for 200 ns in triplicate, and the RMSD plots indicated that all replicates reached equilibrium (Figure 5A). Snapshot analysis revealed similar findings between the replicates, with the ligand maintaining its position within the CRD, as seen in the intermolecular H-bond plot over the course of the simulation (Figure 5B). Therefore, a single trajectory was used in subsequent analysis.

**Figure 6:**
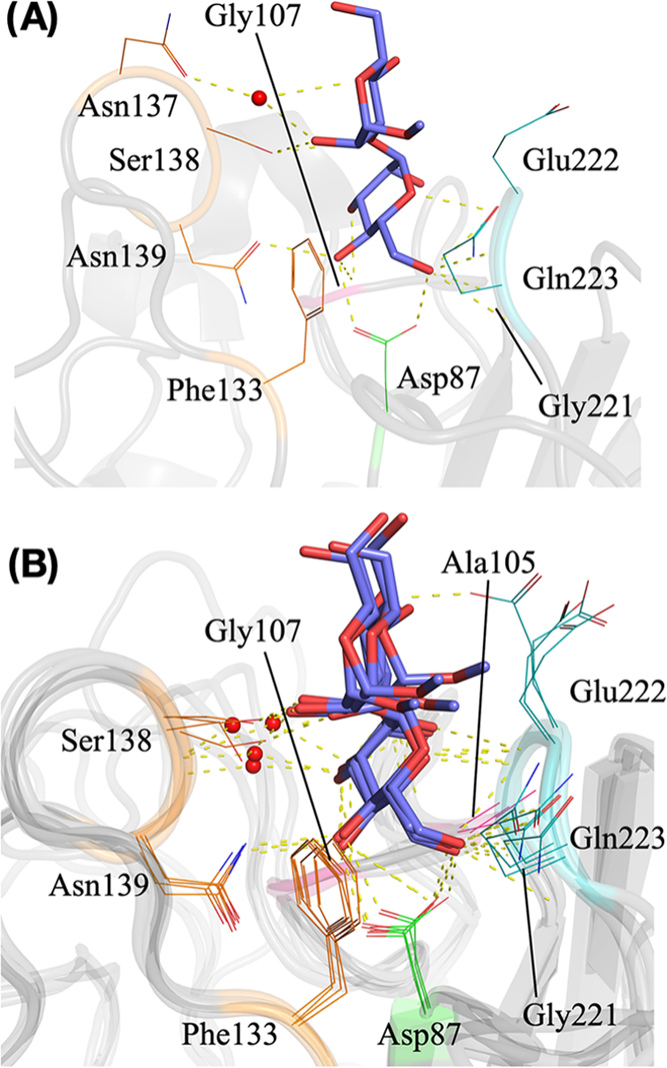
Carbohydrate-binding structural analysis. (A) Lectin–carbohydrate complex as seen in the crystal structure. (B) Overlapped snapshots of representative frames over the course of the molecular dynamics simulation. The structure is shown as ribbons with interacting residues represented as lines in different colors: green for residues in loop A, pink for loop B, orange for loop C, and cyan for loop D. MDM is represented as sticks with carbons in dark blue. Water molecules are shown as spheres in red with yellow dashes representing hydrogen bonds.

Similar to other mannose-specific legume lectins, the binding between the lectin and MDM primarily involves one mannosyl moiety (nonreducing end), with the reducing-end mannose (methylated mannosyl) protruding away from the CRD. The interaction between CML and the nonreducing-end mannose residue inside the CRD involves the following residues: Asp87, Ala105, Gly107, Phe133, Gly221, Glu222, and Gln223 (Figure 6). The carbohydrate interaction of CML also includes two high-frequency (>80%) water bridges seen in the MD simulation and an additional one observed only in the crystal structure, which is likely to be random since it is not seen in both monomers. The most important interactions occur between Asp87 and the O4 and O6 atoms of the ligand, combined with the bond between Asn139 and O4 and the CH–π stacking interaction with Phe133. This positions the ligand to form H-bonds involving the O3 atom of the nonreducing-end mannose residue and the backbones of Gly107, O5 and the backbone of Glu222, and finally O6 and the backbones of Gly221 and Gln223. The two observed water bridges enable distant interactions between Ser138 and the O3 atom of the nonreducing-end mannose residue, as well as Ala105 and the O3 and O4 atoms of the ligand. Low-frequency (<20%) interactions were seen between Ser138 and O1 and Gly221 and O2 of the nonreducing-end mannose residue.

For interactions involving the reducing-end mannose residue, Ser138 forms direct interactions with the O2 of the methylated mannosyl and a low-frequency interaction was seen between Glu222 and the O4 atom.

The overall binding profile of CML follows the same principles that guide other mannose-specific legume lectins from plants. The CRD of CML, like other legume lectins, is formed by four loops, conventionally named loops A, B, C, and D, where the interacting residues are located.^36^ CML binding involves Asp87 in loop A combining with Asn139 and Phe133 in loop C, with the latter being held in place by the metal-binding site. The H-bonds formed by these residues are conserved in virtually all legume lectins and are the main determinants of binding.^37^

Two other loops are important for carbohydrate binding, with loop D being particularly significant. The determinants of specificity in loop D depend more on its size than its composition.^38,39^ Loop C contains an important glycine residue, Gly107, in CML, which interacts with the O3 atom at the mannosyl of the nonreducing end of MDM. The loop D of the CML includes Gly221, Glu222, and Gln223. The main interactions with residues of loop D occur through the backbone, allowing different lectins to have varied residues without significantly affecting binding. CML has the shorter version of loop D, which is common to all mannose-specific lectins.

These properties are common to most mannose-specific legume lectins. However, CML distinguishes itself from other lectins in a few ways rarely reported outside of lectins from the same tribe of plants. These include a large polar residue (Glu222) in the middle of loop D, likely part of the extended binding site of the lectin, similar to Arg228 in loop B of *Canavalia ensiformis* lectin, ConA.^40^ Additionally, a serine residue in loop C forms consistent interactions with the O3 atom of the ligand through a water bridge. Most interestingly, a water bridge was noted between the backbone of Ala105 on loop B and the O2 and O3 atoms at the mannosyl of the nonreducing end of the ligand. This, along with low-frequency interactions between O2 and the loop D, may be a major factor explaining the higher affinity of CML toward mannosides compared to glucosides.^41^

The current work also investigated interactions taking place outside of the monosaccharide binding site, and this resulted in the identification of a few key interactions. These include Ser138 and Glu222 interacting with the methylated mannosyl of the reducing end. This aligns with findings from similar lectins.^23,38^ and, by sequence similarity, it can be suggested that the main residues from the extended binding site are located in loops C and D, including Asn145, Ser146, and Glu222. The presence of these residues and the binding profile seen for MDM suggest a preference for asymmetric complex glycans over high-mannose glycans, although it is likely that both are strong binders.^42,43^

### 3.3 Antiglioma Activity of CML

We have previously shown that lectins with glucose/mannose affinity from Diocleinae subtribe species, such as ConBr, ConGf^13−15^ and DvL, DSL^44,45^ impair glioma cell viability and migration, as well as induce autophagy. In the present study, we evaluated the capacity of CML, a lectin that also demonstrated mannose affinity, to modulate these parameters in C6 glioma cells.

**Figure 7:**
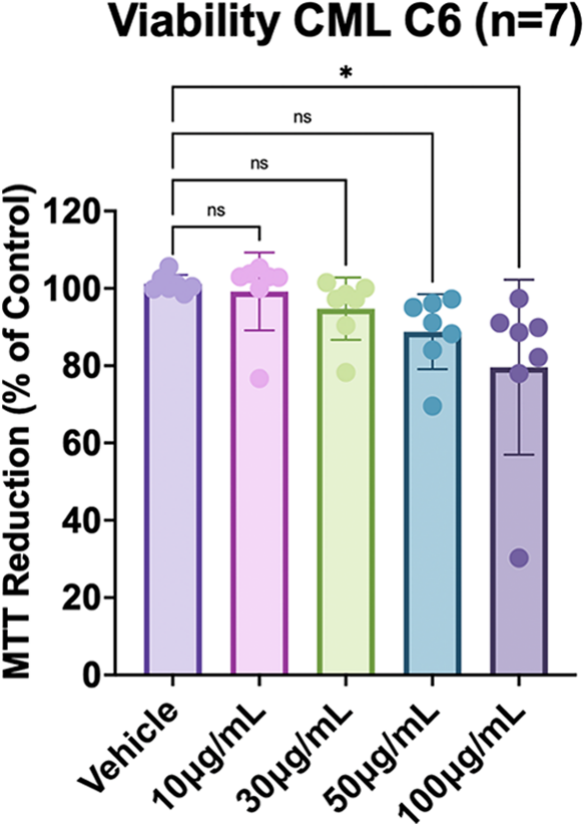
CML decreased C6 glioma cell viability. C6 glioma cells were incubated for 24 h with the vehicle (HEPES-saline buffer; control) or CML at concentrations of 10, 30, 50, and 100 μg/mL. Cell viability was measured by the MTT assay. Seven independent experiments were performed in triplicate. Data quantification was expressed as a percentage of control (considered 100%), and the values are presented as mean ± SEM. Statistical significance was analyzed by one-way ANOVA followed by Tukey’s post-hoc test. *p < 0.05 as compared to the vehicle (control).

The activity of CML (10–100 μg/mL) on glioma cell viability was evaluated by an MTT assay. The results obtained showed that CML decreased tumor cell viability significantly only in the highest concentration tested (100 μg/mL) after 24 h treatment (Figure 7).

**Figure 8:**
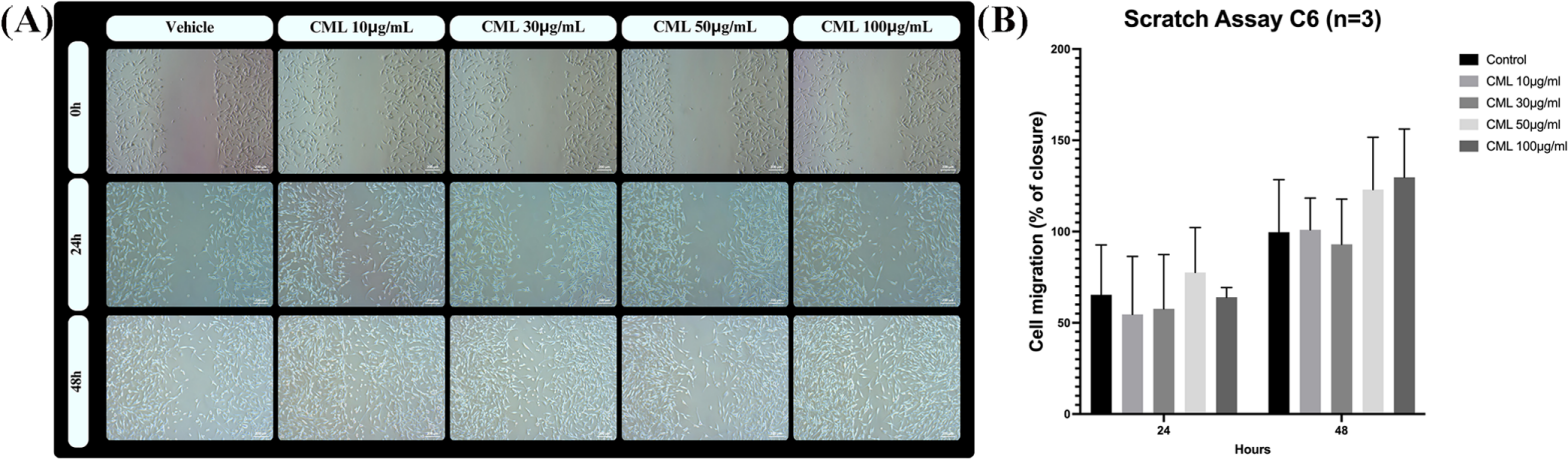
CML did not alter the C6 glioma cell migration. C6 glioma cells were grown for 24 h until confluence. A linear area of attached cells in each well was then removed with a pipet tip (P200), producing a wound. Thereafter, the medium was removed and serum-free DMEM with the vehicle (control) or CML (10, 30, 50, and 100 μg/mL) was added. (A) Images immediately after scratching (time 0) and after 24 and 48 h in response to each treatment. Cells were visualized under light microscopy by an inverted NIKON eclipse T2000-U microscope (5×). The bars represent 200 μm. (B) Quantification of cell migration that was performed by measurement of wound closure and expressed as a percentage related to control cells considered as 100% closure (at time point 48 h). Data (*N* = 3) are presented as mean ± SEM. Statistical significance was analyzed by two-way ANOVA, and no difference was found in the ratio of cell migration between CML and the vehicle.

Unlike other lectins, CML (10–100 μg/mL) was unable to alter C6 glioma cell migration and proliferation, as evaluated by the scratch assay. Therefore, as presented in the representative panel (Figure 8A) and the quantification shown in Figure 8B, the closure ratio of the wound, after 24 and 48 h, was similar to that in the control- (vehicle) and CML-treated cells.

**Figure 9:**
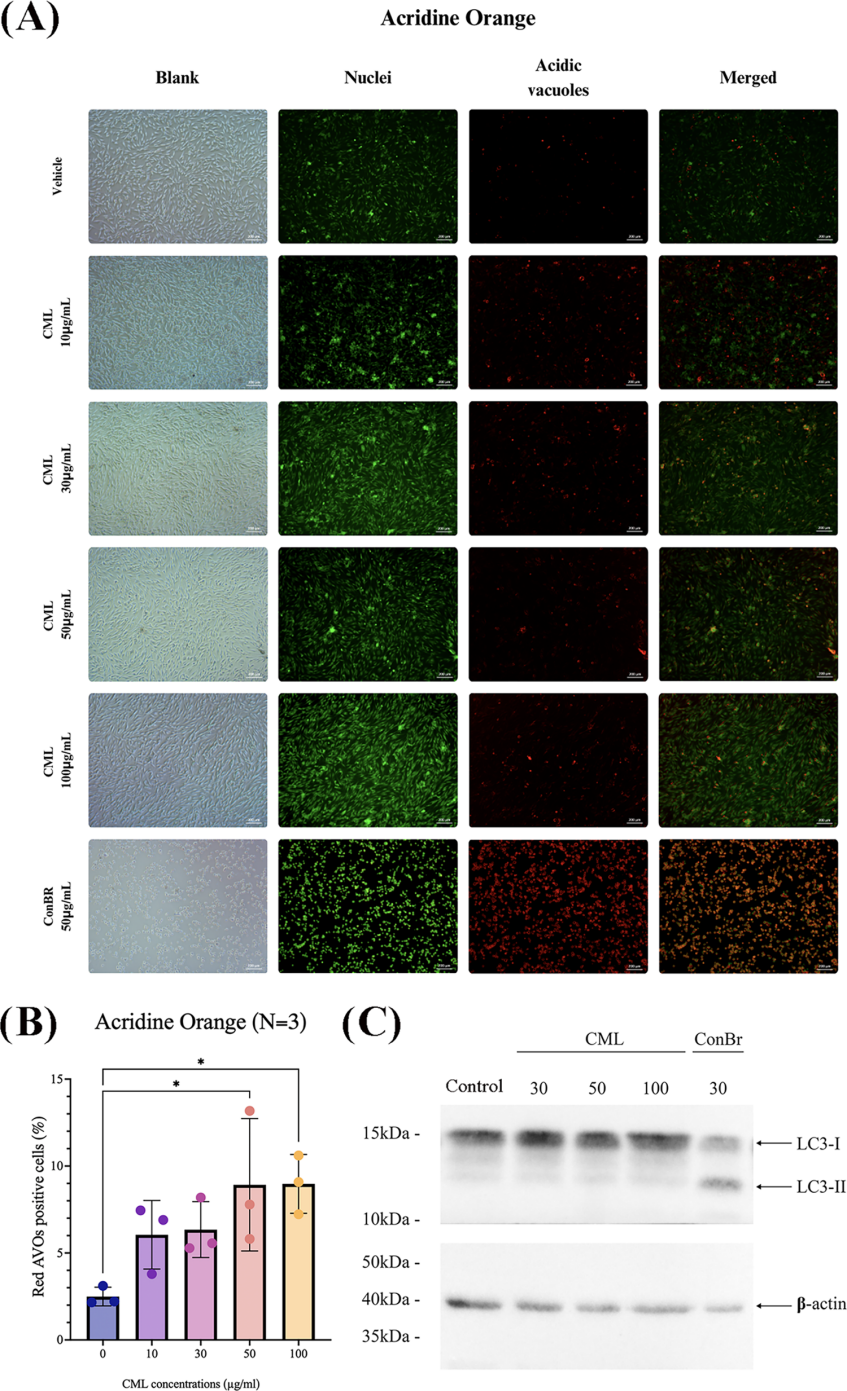
CML induces autophagy in C6 glioma cells. (A and B) Cells were treated for 24 h with the vehicle (HEPES-saline Buffer; control) or CML at concentrations of 10, 30, 50, and 100 μg/mL, as well as with ConBr 50 μg/mL. Thereafter, cells were stained with acridine orange. (A) Representative image of cells visualized by light microscopy and by acridine orange staining. Chromatin (CR) and acidic vesicular organelles (AVOs) were detected in green and red channels, respectively. The bars represent 200 μm. (B) For quantification of AVOs, the data are expressed as arbitrary units, and the values are presented as mean ± SEM. Statistical significance was analyzed by one-way ANOVA, followed by Tukey’s post-hoc test. *p < 0.05 as compared to the vehicle (control). (C) Cells (500,000 cells/well) were treated for 24 h with the vehicle (HEPES-saline Buffer; control) or CML at concentrations of 30, 50, and 100 μg/mL, as well as with ConBr 30 μg/mL. After treatment, cells were homogenized with the sample buffer, and 50 μg of protein/track was electrophoresed, followed by electrotransfer into the nitrocellulose membrane. The panel shows a representative Western blot membrane (*n* = 3) of LC3I (16 kDa) and LC3II (14 kDa). B-actin is shown as the loading control.

The induction of autophagy in tumor cells has been described for many lectins.^12,14,46,47^ Accordingly, we evaluated the autophagic process after CML treatment using the acridine orange assay and LC3II analysis (Figure 9). The representative panel and the quantification of AVOs are presented in Figure 9A,B, respectively, showing that CML at higher concentrations of 50–100 μg/mL induces an increment in the levels of acidic vesicular organelles (AVOs) formation. Moreover, CML promoted a slight increase in the level of LC3II, a protein involved in autophagy processes (Figure 9C). Taken together, these results suggest the potential for CML to stimulate autophagy in C6 glioma cells. However, compared with ConBr (30–50 μg/mL), a lectin previously described to induce autophagy and glioma cell death,^13,15^ it is noteworthy that the increment of AVOs (Figure 9A,B), or the lipidated form of LC3 (known as LC3II), by CML was lower than that observed for ConBr. Moreover, unlike ConBr, CML did not modify the cell morphology nor did it induce the loss of cell adhesion, as shown by light microscopy (Figure 9A). Noteworthy, our results concerning antiglioma activity of CML were more similar to ConV, a legume lectin from *Canavalia virosa* that displayed cytotoxicity only at a high concentration of 100 ug/mL.^48^

Although CML and ConA-like proteins, such as ConBr and ConGf, are specific for mannose and have similar binding modes, they have distinct effects on glioma cells. The antiglioma activity of CML is low compared to the strong activity of ConA-like proteins. These different effects may be attributed to the oligomeric states of the proteins. That is, although both proteins can recognize and bind to mannosides, the tetrameric state of ConA-like proteins affords stronger multivalency, which likely allows them to better interact with structurally complex glycans found on the cell surface. Previous studies have shown that the tetrameric state increases the affinity for these complex glycans, which, in turn, facilitates more effective binding.^40,49,50^ In contrast, CML forms dimers and has fewer simultaneous binding sites. As a result, it does not interact as well with glycans on cells. Its ability to induce aggregation of cellular receptors and initiate signaling cascades related to cell death or growth inhibition is also impaired by this lower multivalency. These signaling cascades are essential for an effective antiglioma response.^13,14^ Therefore, even with similar molecular bases and mannose specificity, the differential oligomeric state between CML and ConA-like proteins is a factor that may determine the effects on glioma cells.

**Figure 10:**
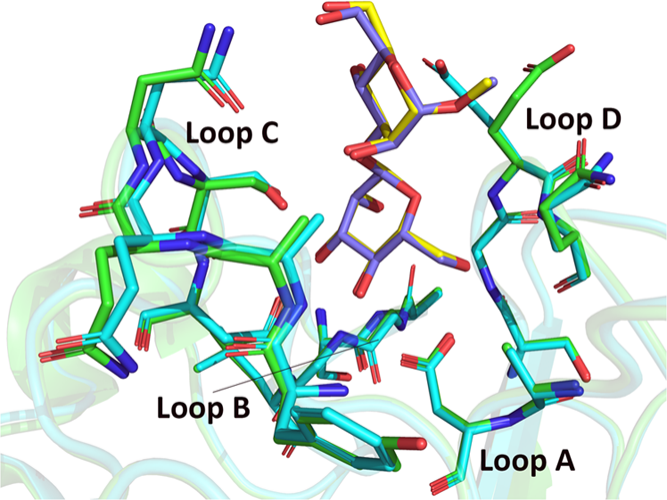
Superposition of the three-dimensional structures of CML (green) and PELa (cyan), highlighting the residues of the four loops that form the carbohydrate-recognition domain. MDM is in stick format with carbons colored yellow (CML) and purple (PELa).

In addition, CML likely has a glycan specificity distinct from that of ConA. CML shares 88% identity with PELa and exhibits a strong similarity in the amino acid composition of the four loops responsible for forming the carbohydrate-recognition domain (Figure 10). Therefore, it can be inferred that the glycan specificity of CML is closer to that of PELa than to that of ConA. Classical mannose-specific legume lectins, such as ConA, preferentially bind high-mannose-type *N*-glycans, with reduced affinity for complex *N*-glycans and minimal interaction with asymmetric *N*-glycans. In contrast, PELa demonstrates a unique specificity for asymmetric glycans, clearly distinguishing it from ConA-like lectins.^43^

The use of various *N*-glycosylation inhibitors demonstrated that the proliferation and attachment of C6 cells are associated with the presence of surface glycoproteins rich in oligomannosides and hybrid *N*-glycans. Conversely, the absence of *N*-glycans, glucosyl-oligomannosides, or glycoproteins significantly reduced both proliferation and attachment.^9,51^ The abundant presence of high-mannose *N*-glycans, essential for cell multiplication, justifies the strong antiglioma activity of ConA on these cells, in contrast to that of CML. Although the glycosylations present in C6 glioma cells are not yet fully understood, it is possible to suggest that the differences in specificity between CML and ConA-like lectins may directly impact antiglioma activity. The composition of glycans on the surface of C6 cells could allow for more favorable interaction with ConA compared to CML. Despite not knowing the exact glycosylation profile, the literature reveals that cancer cells exhibit distinct glycosylation profiles compared to normal cells, with a notable increase in high-mannose glycans and a decrease in complex glycans. This altered glycosylation pattern is observed across various cancer types, including breast, colorectal, lung, and prostate cancers.^52^

## 4 Conclusions

This study provided a detailed analysis of the three-dimensional structure of *C. microchaete* lectin and its interactions with carbohydrates, particularly methyl-mannose-α1,3-mannose. Using X-ray crystallography, the lectin was found to adopt a β-sandwich fold and form canonical dimers but not tetramers, which appears to limit its functional efficacy in the tested biological context. Functionally, the lectin exhibited only modest cytotoxic effects on C6 glioma cells at high concentrations with no observed impact on cell migration or morphology. Although it was capable of inducing autophagy, its effects were significantly weaker than those of the ConA-like lectins. This suggests that structural features beyond mannose specificity, such as the ability to form tetramers and interact effectively with cellular glycans, are critical for robust antiglioma activity. Overall, these findings indicate the importance of both structural and functional characteristics in determining the biotechnological potential of lectins and highlight the need for further studies to explore how specific glycan interactions influence cellular signaling and cytotoxicity in gliomas.

## Data Availability

The data supporting the findings of this study are available from the corresponding authors upon reasonable request. Crystallography data can be accessed in the Protein Data Bank under Accession Code 9C4I.

## Supporting Information Available

Images related to additional structural analyses of *Centrolobium microchaete* seed lectin, including glycosylation site details, dimer stabilization interactions, metal-binding coordination, ligand interaction mapping, and structural comparisons with related lectins (PDF)
