# Supplementary material for: Crystallographic Structure and Antiglioma Potential of Centrolobium microchaete Seed Lectin
Source: ACS Omega. 2025 Jan 27;10(5):4686–98. doi: 10.1021/acsomega.4c09145 (PMC11822720; doi:10.1021/acsomega.4c09145)

Electronic Supplementary Information

Crystallographic structure and anti-glioma potential of *Centrolobium microchaete* seed lectin

Benildo Sousa Cavada[1], Vanir Reis Pinto-Junior[1], Francisco Edilcarlos Oliveira Lima[1], Valeria Maria Sousa Ferreira[1], Messias Vital Oliveira[1], Vinicius Jose Silva Osterne[1,2], Nicole Sartori[3]; Ana Carolina dos Santos[3], Rodrigo Bainy Leal[3,*] and Kyria Santiago Nascimento[1,*]

[1]*Department of Biochemistry and Molecular Biology, BioMolLab, Federal University of Ceara, Fortaleza 60020-181, CE, Brazil*

[2]*Laboratory of Biochemistry and Glycobiology, Department of Biotechnology, Ghent University, 9000 Ghent, Belgium*

[3]*Department of Biochemistry and Postgraduate Program in Biochemistry, Center for Biological Sciences, University Campus, Federal University of Santa Catarina, Florianópolis 88040-900, SC, Brazil*

[*]Authors to whom correspondence should be addressed (R.B.L., rbleal@gmail.com; K.S.N., kyriasantiago@ufc.br).

Figure S1. GlcNAc residue present in the *N*-glycosylation site of CML at residue 119. A) Monomer 1; B) Monomer 2.

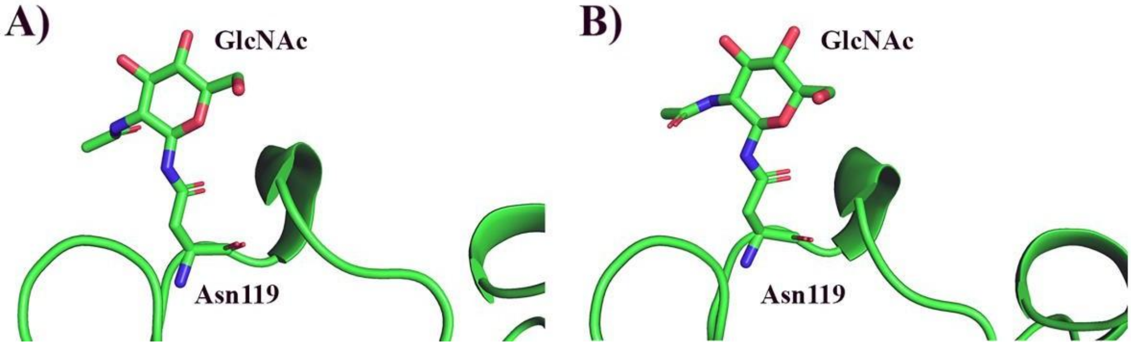

Figure S2. Amino acids are involved in interactions at the dimeric interface responsible for stabilizing the CML canonical dimer. Monomer 1 is in green and monomer 2 is in blue.

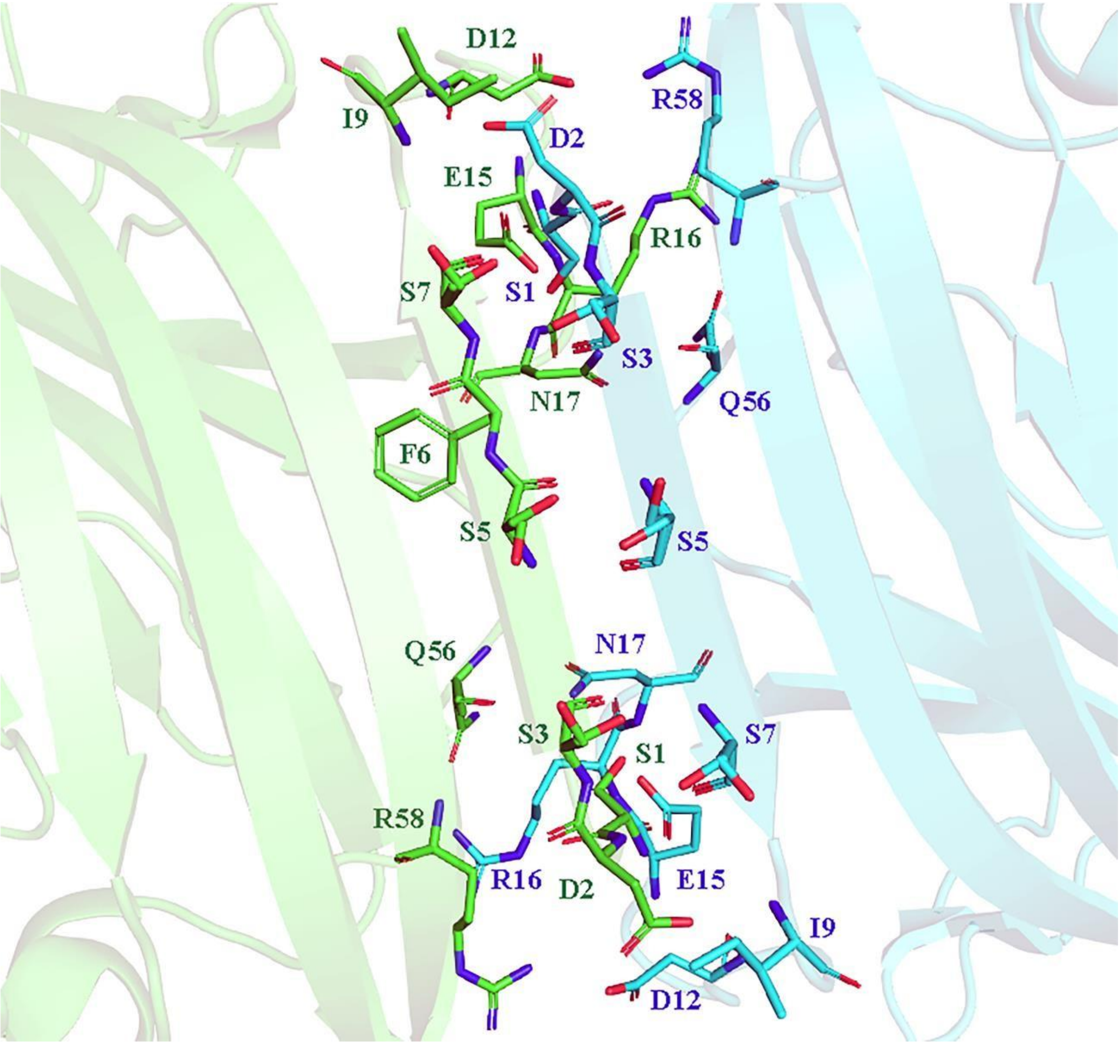

Figure S3. The metal binding site of CML with octahedral coordination interactions around A) calcium (green) and B) manganese (purple) ions.

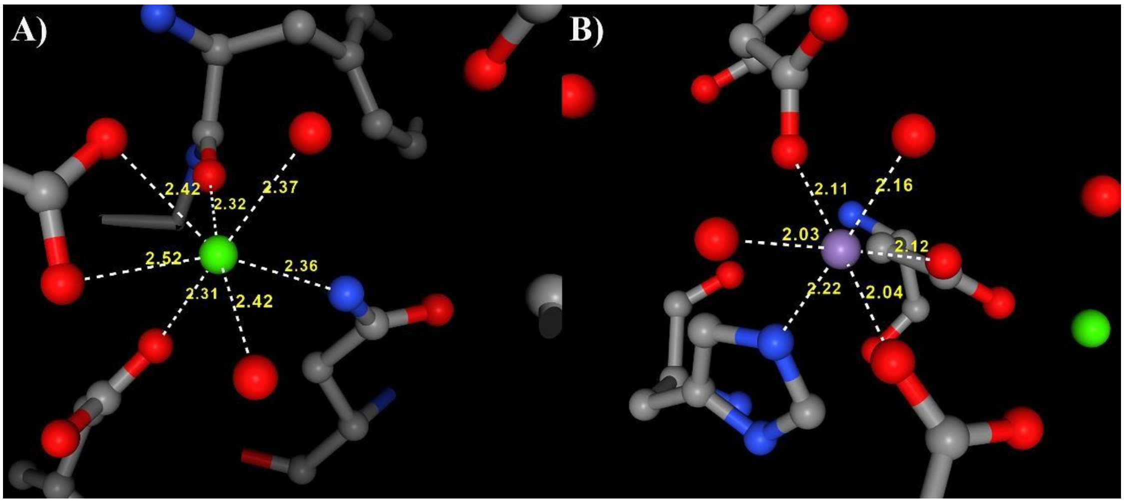

Figure S4. Supplementary Figure X2. LIGPLOT representations of hydrogen and hydrophobic bonds around the MDM. A) CRD of monomer 1; B) CRD of monomer 2.

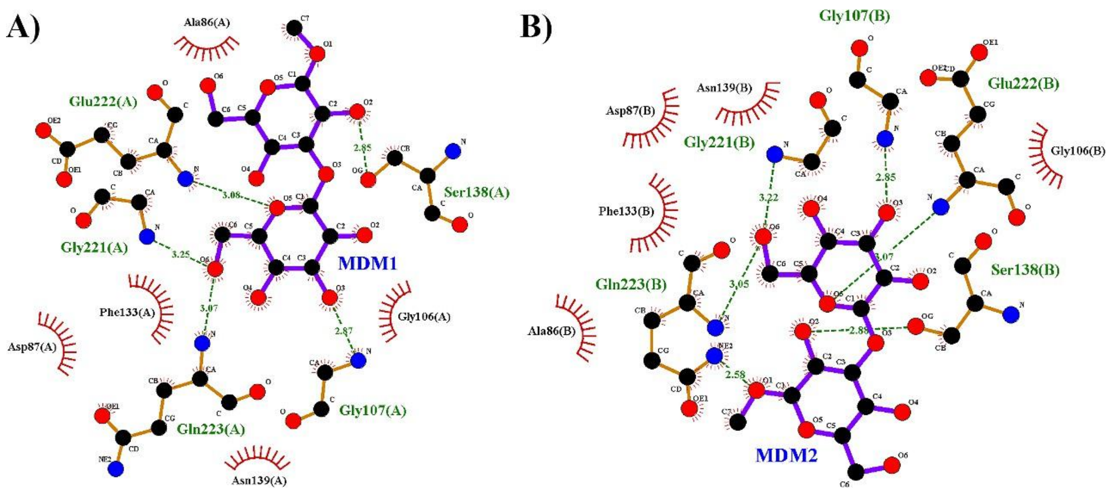

Figure S5. Cartoon representation of the CML superposition (in green) with CTL (PDB id: 5EYY, in red), PELa (PDB id: 5U38, in blue) and PAL (PDB id: 1Q8P, in yellow).

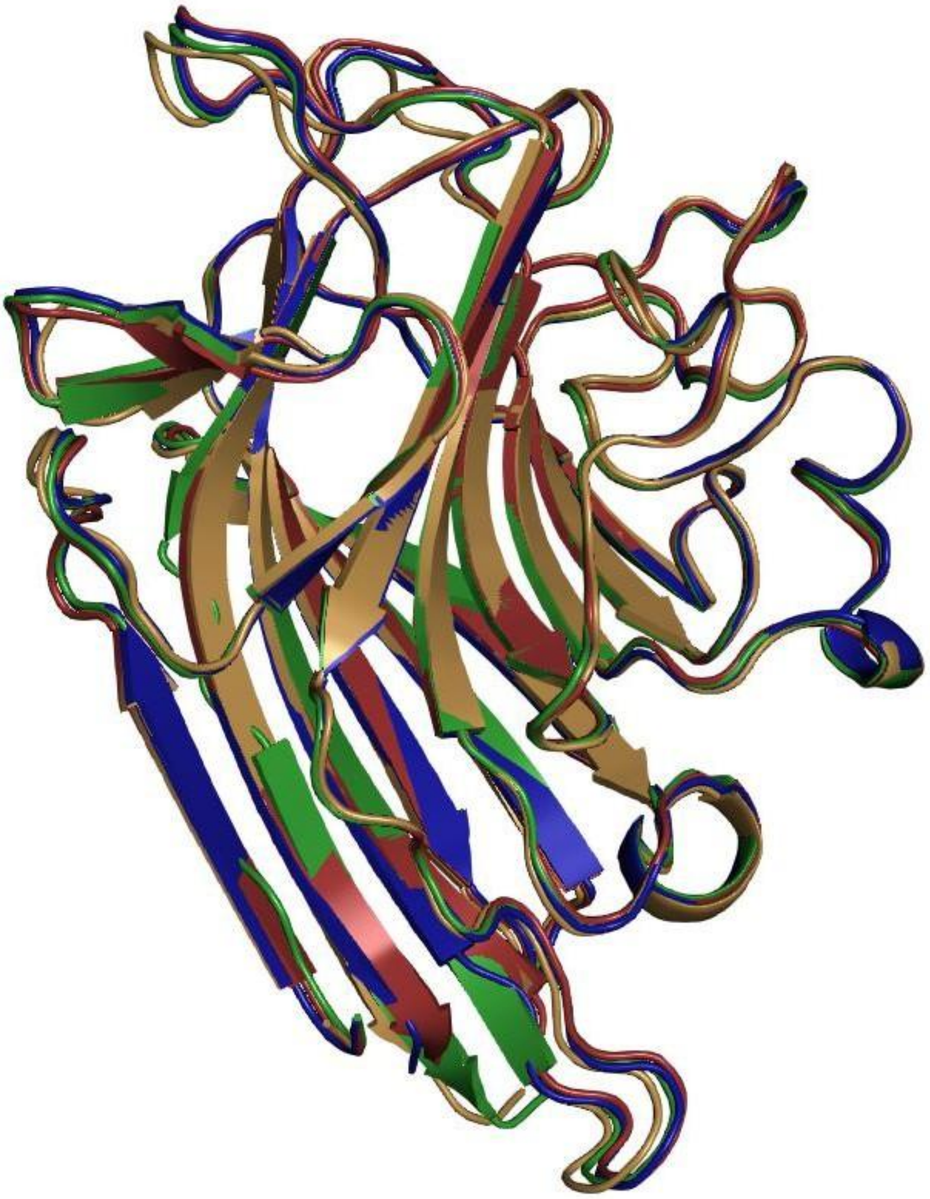

Supplement: Supplementary file 1 — ao4c09145_si_001.pdf [file ao4c09145_si_001.pdf]
